# Entropy-Based Evidence Functions for Testing Dilation Order via Cumulative Entropies

**DOI:** 10.3390/e27121235

**Published:** 2025-12-05

**Authors:** Mashael A. Alshehri

**Affiliations:** Department of Quantitative Analysis, College of Business Administration, King Saud University, Riyadh 11362, Saudi Arabia; mealshehri@ksu.edu.sa

**Keywords:** dilation order, cumulative residual entropy, cumulative entropy, evidence functions, statistical inference, non-parametric methods, HNBUE, L-statistics, test power

## Abstract

This paper introduces novel non-parametric entropy-based evidence functions and associated test statistics for assessing the dilation order of probability distributions constructed from cumulative residual entropy and cumulative entropy. The proposed evidence functions are explicitly tuned to questions about distributional variability and stochastic ordering, rather than global model fit, and are developed within a rigorous evidential framework. Their asymptotic distributions are established, providing a solid foundation for large-sample inference. Beyond their theoretical appeal, these procedures act as effective entropy-driven tools for quantifying statistical evidence, offering a compelling non-parametric alternative to traditional approaches, such as Kullback–Leibler discrepancies. Comprehensive Monte Carlo simulations highlight their robustness and consistently high power across a wide range of distributional scenarios, including heavy-tailed models, where conventional methods often perform poorly. A real-data example further illustrates their practical utility, showing how cumulative entropies can provide sharper statistical evidence and clarify stochastic comparisons in applied settings. Altogether, these results advance the theoretical foundation of evidential statistics and open avenues for applying cumulative entropies to broader classes of stochastic inference problems.

## 1. Introduction

Modern statistical inference increasingly emphasizes the quantification of statistical evidence, functions that measure the support that observed data provide for competing models or for specific structural features of the underlying generating process. Classical information-theoretic criteria, such as Kullback–Leibler divergence and model selection scores (AIC, BIC), are powerful for assessing overall fit, but many applications in reliability, finance, and insurance require evidence about distributional variability, stochastic comparisons, and ordered structural properties, not only global fit.

Stochastic orders provide a rigorous language for such comparisons. They rank distributions by characteristics such as location, variability, and risk, and are widely used in reliability, actuarial science, economics, and finance. Among these orders, the dilation order is central because it ranks random variables by dispersion, thereby offering a principled evidential framework for assessing relative variability. This is particularly valuable when analyzing heavy-tailed or complex data, where traditional dispersion measures such as variance can be insensitive or misleading.

The dilation order therefore plays a crucial role in applications where understanding differences in spread rather than central tendency is essential. For example, in survival analysis, finance, and insurance, practitioners often seek evidence regarding whether one population exhibits greater dispersion than another, rather than whether their means differ. Developing non-parametric tools tailored to this question is both theoretically important and practically necessary, especially when model assumptions cannot be guaranteed. Recall that a random variable Y is more dispersed in the dilation order than a random variable X (denoted by *X* ≤_*dil*_ *Y*) if

(1) E[ϕ(X−E(X))]≤E[ϕ(Y−E(Y))],

for every convex function ϕ for which the expectations exist [1]. This formalizes that Y displays greater dispersion than X. It is immediate that *X* ≤_dil_ *Y* implies Var(X)≤Var(Y), but variance induces a total order and is therefore less informative than the partial order provided by dilation. For background and related variability orders, see [2,3,4]. In parallel, measuring uncertainty via entropy functions has become an active area in statistics and information theory. Shannon’s [5] differential entropy for a nonnegative random variable X with density (pdf) f and distribution function (cdf) F is defined by:

(2) $$H\left(X\right)=-\int_{0}^{\infty }\;f\left(x\right)\log f\left(x\right)dx,$$

where “log(⋅)” means natural logarithm. The quantity H(X) is location-free since X and X+b share the same differential entropy for any b>0. Thus, negative values of b are allowed whenever $\min\left(X\right)+b>0.$ However, expression (2) has well-known limitations as a continuous analog of discrete entropy, which has motivated the development of alternative measures, including weighted entropy and residual/past entropy variants [6,7]. The cumulative residual entropy (CRE), defined by:

(3) $$CRE\left(X\right)=-\int_{0}^{\infty }\;\;S\left(x\right)\log S\left(x\right)dx=\int_{0}^{\infty }\;\;S\left(x\right)\Lambda \left(x\right)dx,$$

where S(x)=P(X>x) denotes the survival function, and

(4) $$\Lambda (x)=-logS(x)=\int_{0}^{x}\;\;\frac{f\left(u\right)}{S\left(u\right)}du,x>0,$$

denotes the cumulative hazard function [8]. The CRE effectively characterizes information dispersion and has numerous applications in reliability and aging analysis [9,10,11,12,13]. From an evidential perspective, CRE functions not only as a measure of uncertainty but also as a tool for drawing statistical evidence about variability and aging behavior.

The cumulative entropy (CE), an alternative probability-based measure associated with inactivity time, is obtained by replacing the pdf with the cdf in Shannon’s definition

(5) $$CE\left(X\right)=-\int_{0}^{\infty }\;\;F\left(x\right)\log F\left(x\right)dx=\int_{0}^{\infty }\;\;F\left(x\right)T\left(x\right)dx,$$

where

(6) $$T(x)=-logF(x)=\int_{x}^{\infty }\;\;\frac{f\left(u\right)}{F\left(u\right)}du,x>0,$$

denotes the cumulative reversed hazard function [14]. Because the logarithmic argument is a probability, both CRE(X) and CE(X) are nonnegative; by contrast, H(X) may be negative for continuous variables. Moreover, $CE\left(X\right)=0$ if and only if X is degenerate, underscoring its role as a measure of uncertainty. Properties of CE and its dynamic version for past lifetimes establish useful connections to characterization and stochastic orderings, reinforcing its value as a functional tool for comparing distributions. Several recent developments on cumulative entropies and their applications are presented in [15,16,17,18,19,20].

Within the evidential framework, an evidence function is designed to estimate a clearly defined target that represents the scientific contrast of interest. The quality of an evidence function is evaluated through desiderata such as consistency, interpretability, and its explicit treatment of uncertainty. Recent work has clarified the distinction between statistical evidence, and Neyman–Pearson (NP) testing and has formalized the link between an evidence function and its associated target in applied analysis [21,22,23,24,25].

While evidential statistics treats statistical evidence as a continuous and independent measure of support for one hypothesis over another, clearly separating evidence from belief and decision, most applications to date have relied on parametric models (e.g., Cahusac [26], Dennis et al. [25], and Powell et al. [27]). In contrast, our approach provides the first fully non-parametric construction of an evidence function, thereby extending the evidential framework beyond parametric assumptions. Traditional tests often fail to quantify the relative support for competing scientific claims; for example, a non-significant *p*-value does not imply equality of variances but merely indicates insufficient evidence against the null hypothesis. In contrast, evidential functions such as the entropy-based estimators proposed in this work are continuous, portable, and accumulable, providing estimates of well-defined inferential targets. Importantly, even under model misspecification, evidential methods preserve interpretable error properties, whereas classical tests can become increasingly misleading as data size grows (see Taper et al. [21] for further discussion).

This paper develops entropy-based evidence functions for the dilation order. We define two quantities, ${\hat{D}}_{1}(n,m)$ (CRE-based) and ${\hat{D}}_{2}(n,m)$ (CE-based), as evidence estimators of targets $D_{1}(X,Y)$ and $D_{2}(X,Y)$ that quantify the degree of stochastic variability between two distributions. Our primary framing is evidential: ${\hat{D}}_{1}(n,m)$ and ${\hat{D}}_{2}(n,m)$ are constructed and analyzed as evidence functions; NP-style tests are presented only as optional, secondary adaptations for decision-making. This reframing yields two concrete extensions: (i) model-to-process feature evidence, comparing models to generating processes with respect to dispersion/variability; and (ii) process-to-process evidence, comparing distinct generating processes by their distributional features using CRE and CE.

By framing ${\hat{D}}_{1}(n,m)$ and ${\hat{D}}_{2}(n,m)$ as evidence functions for the dilation order, this study aligns with the modern evidential paradigm. The objective is not to reject or accept a null hypothesis of equal dispersion, but to quantify the degree to which the observed data support one distribution as more dispersed than another. This approach is robust to heavy-tailed behavior, invariant to location shifts, and firmly grounded in information-theoretic principles. Moreover, the evidential perspective naturally accommodates comparisons between models and empirical reality, or between two observed processes, without requiring either model to be strictly true. This property represents a critical advantage in fields such as reliability, finance, and insurance, where all models are inherently approximations.

Methodologically, we derive large-sample distributions for the proposed functionals, provide practical nonparametric estimators, and evaluate performance via Monte Carlo experiments spanning light- and heavy-tailed families. A real-data application (survival times) illustrates interpretability and robustness. Compared with existing dilation-order procedures [4,28,29], the proposed approach is straightforward to implement, computationally efficient, and often competitive in power, particularly under heavy tails.

The remainder of this paper is organized as follows. Section 2 presents the theoretical foundations of CRE and CE under the dilation order. Section 3 introduces the proposed evidential functionals and investigates their asymptotic properties. Furthermore, it evaluates the accuracy of the proposed measures using a Monte Carlo simulation and demonstrates the methodology through a real-data application. Finally, Section 4 summarizes the main findings and outlines potential directions for future research.

Throughout this paper, we consider the random variables to be absolutely continuous, and we assume that all integrals and expectations exist whenever they are mentioned in the text.

## 2. Preserving the Dilation Order via Cumulative Entropies

This section investigates how CRE and CE can serve as evidence functions for assessing the dilation order. A key advantage of CRE lies in its connection to the mean residual life (MRL) function, m(x)=E(X−x∣X>x). It has been shown that $CRE\left(X\right)=E\left[m\left(X\right)\right]$, a relationship that underscores the relevance of CRE in reliability theory, where the MRL function is widely used to describe system aging [10]. Similarly, CE is closely related to the mean inactivity time (MIT) function, $\tilde{m}(x)=E(x-X|X\leq x)$; in particular, $CE(X)=E[\tilde{m}(X)]$ [14]. These connections highlight the role of CRE and CE not only as measures of uncertainty but also as practical adequacy measures for quantifying variability. We now turn to the relationship between the dilation order of two random variables and the ordering of their CRE and CE. Recall that for a random variable X with cdf F, the quantile function is defined by $F^{-1}(u)=inf\{x:F(x)\geq u\}$ for u∈(0,1).

**Theorem** **1.** *Let* X *and* Y *be two absolutely continuous nonnegative random variables with respective finite means* E(X) *and* E(Y) *and with pdfs* f *and* g *and cdfs* F *and* G*, respectively. Then,*

*(i)*  *if* *X* ≤_*dil*_ *Y, then* CRE(X)≤CRE(Y).
*(ii)*  *if* *X*≤ _*dil*_ *Y, then* CE(X)≤CE(Y).

**Proof.**  (i) Given that $E(X|X>x)=\frac{1}{S(x)}\int_{x}^{\infty }\;uf(u)du$, and m(x)=E(X∣X>x)−x, along with the relation CRE(X)=E[m(X)] and Equation (4), we can rewrite CRE(X) as:

(7) $$\begin{array}{ll} CRE\left(X\right) & =\int_{0}^{\infty }\;\;E\left(X|X>x\right)f\left(x\right)dx-E\left(X\right)\\ & =\int_{0}^{\infty }\;\;\frac{1}{S(x)}\int_{x}^{\infty }\;\;uf\left(u\right)duf\left(x\right)dx-E\left(X\right)\\ & =\int_{0}^{\infty }\;\;uf\left(u\right)\int_{0}^{u}\;\;\frac{f\left(x\right)}{S\left(x\right)}dxdu-E\left(X\right)\\ & =-\int_{0}^{\infty }\;\;uf\left(u\right)\log S\left(u\right)du-E\left(X\right)\\ & =-\int_{0}^{1}\;\;F^{-1}\left(u\right)\log\left(1-u\right)du-E\left(X\right), \end{array}$$

where the final equality follows from the substitution u=F(x). An analogous identity holds for CRE(Y). Recall from Theorem 3.A.8 of [2] that $X\leq_{dil}Y$ implies

$$\frac{1}{1-p}\int_{p}^{1}\;\left[F^{-1}(u)-G^{-1}(u)\right]du\leq \int_{0}^{1}\;\left[F^{-1}\left(u\right)-G^{-1}\left(u\right)\right]du,\;for\;all\;p\in [0,1].$$

Since $E(X)=\int_{0}^{1}\;F^{-1}(u)du$ and $E(Y)=\int_{0}^{1}\;G^{-1}(u)du$, integrating both sides of the inequality over [0,1] and applying Fubini’s theorem yields

$$\begin{array}{ll} E(X)-E(Y) & \geq \int_{0}^{1}\;\;\frac{1}{1-p}\int_{p}^{1}\;\;\left[F^{-1}(u)-G^{-1}(u)\right]dudp\\ & =\int_{0}^{1}\;\;\left[F^{-1}(u)-G^{-1}(u)\right]\int_{0}^{u}\;\;\frac{1}{1-p}dpdu\\ & =-\int_{0}^{1}\;\;\left[F^{-1}(u)-G^{-1}(u)\right]log(1-u)du\\ & =\int_{0}^{1}\;\;G^{-1}\left(u\right)\log\left(1-u\right)du-\int_{0}^{1}\;\;F^{-1}\left(u\right)\log\left(1-u\right)du. \end{array}$$

This implies

$$-\int_{0}^{1}\;F^{-1}\left(u\right)\log\left(1-u\right)du-E\left(X\right)\leq \int_{0}^{1}\;G^{-1}\left(u\right)\log\left(1-u\right)du-E\left(Y\right),$$

or equivalently, CRE(X)≤CRE(Y), by recalling relation (7).

(ii) Using $E(X|X\leq x)=\frac{1}{F(x)}\int_{0}^{x}\;uf(u)du,\tilde{m}(x)=x-E(X|X\leq x)$, and Equation (6), we can rewrite $CE(X)=E[\tilde{m}(X)]$, as in Part (i), as follows:

(8) $$\begin{array}{ll} CE\left(X\right) & =-\int_{0}^{\infty }\;\;uf\left(u\right)\log F\left(u\right)du+E\left(X\right)\\ & =\int_{0}^{1}\;\;F^{-1}\left(u\right)\log\left(u\right)du+E\left(X\right), \end{array}$$

where the last equality follows from the substitution u=F(x). The same identity holds for CE(Y). Recall from [2] that $X\leq_{\mathrm{dil}\;}Y$ yields

$$\int_{0}^{1}\;\left[F^{-1}(u)-G^{-1}(u)\right]du\leq \frac{1}{p}\int_{0}^{p}\;\left[F^{-1}\left(u\right)-G^{-1}\left(u\right)\right]du,\;for\;all\;p\in [0,1].$$

Integrating both sides of the preceding relation over [0,1] and applying Fubini’s theorem yields

$$\begin{array}{ll} E(X)-E(Y) & \leq \int_{0}^{1}\;\;\frac{1}{p}\int_{0}^{p}\;\;\left[F^{-1}\left(u\right)-G^{-1}\left(u\right)\right]du\;dp\\ & =\int_{0}^{1}\;\;\left[F^{-1}(u)-G^{-1}(u)\right]\int_{u}^{1}\;\;\frac{1}{p}dpdu\\ & =\int_{0}^{1}\;\;G^{-1}\left(u\right)\log\left(u\right)du-\int_{0}^{1}\;\;F^{-1}\left(u\right)\log\left(u\right)du. \end{array}$$

This implies that

$$\int_{0}^{1}\;F^{-1}\left(u\right)\log\left(u\right)du+E\left(X\right)\leq \int_{0}^{1}\;G^{-1}\left(u\right)\log\left(u\right)du+E\left(Y\right),$$

or equivalently CE(X)≤CE(Y) by (8). This completes the proof. □

Before presenting the next theorem, we define the absolute lower Lorenz curve, used in economics to compare income distributions, as

$$A_{X}\left(p\right)=\int_{0}^{p}\;\left[F^{-1}\left(t\right)-E\left(X\right)\right]dt,$$

for all 0≤p≤1, see e.g., [4]. When X is a degenerate random variable, $A_{X}\left(p\right)$ coincides with the horizontal axis. $A_{X}\left(p\right)$ decreases for $0\leq p\leq F(E\left(X\right))$ and increases for $F(E\left(X\right))\leq p\leq 1$, with $A_{X}(0)=A_{X}(1)=0$. Furthermore, $A_{X}\left(p\right)$ is a convex function with respect to p implying $A_{X}\left(p\right)\leq 0$ for all p∈[0,1]. Note that t can also be written as $A_{X^{⋆}}\left(p\right)=\int_{0}^{p}\;F_{X^{⋆}}^{-1}\left(t\right)dt,$ so its convexity in p reflects the convexity of functionals of the demeaned variable $X^{⋆}=X-E[X]$, consistent with the definition of the dilation order given in (1). Moreover, we also define the complete function of the absolute upper Lorenz curve as follows:

$${\bar{A}}_{X}\left(p\right)=\int_{p}^{1}\;\left[F^{-1}\left(t\right)-E\left(X\right)\right]dt,\;for\;all\;0\leq p\leq 1.$$

It follows that

$${\bar{A}}_{X}\left(p\right)+A_{X}\left(p\right)=0,\;for\;all\;0\leq p\leq 1.$$

From Theorem 3.A.8 of [2], we have $X\leq_{\mathrm{dil}\;}Y$ if and only if $A_{X}\left(p\right)\geq A_{Y}\left(p\right)$ or ${\bar{A}}_{X}\left(p\right)\leq {\bar{A}}_{Y}\left(p\right)$ for all 0≤p≤1. Let us check this with an example.

**Example** **1.** *Let* X *and* Y *be random variables following exponential distributions with cdfs* $F\left(x\right)=1-e^{-\lambda_{X}x},\;x>0,\lambda_{X}>0,$ *and* $G\left(x\right)=1-e^{-\lambda_{Y}x},\;x>0,\lambda_{Y}>0,$ *where* $\lambda_{X}>\lambda_{Y}.$ *It is easy to see that*

$$A_{X}\left(p\right)=\frac{1}{\lambda_{X}}\left(1-p\right)\log\left(1-p\right),\;and\;A_{Y}\left(p\right)=\frac{1}{\lambda_{Y}}\left(1-p\right)\log\left(1-p\right),$$

*for* 0<p<1. *Since* $\lambda_{X}>\lambda_{Y}$ *and* $\left(1-p\right)\log\left(1-p\right)$ *is negative for* 0≤p≤1*, we conclude that*

$$\frac{1}{\lambda_{X}}\left(1-p\right)\log\left(1-p\right)\geq \frac{1}{\lambda_{Y}}\left(1-p\right)\log\left(1-p\right),$$

*which means that* $A_{X}\left(p\right)\geq A_{Y}\left(p\right)$ *for all* 0<p<1 *and hence* $X\leq_{dil\;}Y$.

**Remark** **1.** *We recall that the results given in Example 1 are consistent with the fact that for exponential random variables,* $X\leq_{disp\;}Y$ *whenever* $\lambda_{X}>\lambda_{Y}$ *(see [2] for the definition of the dispersive order* $\leq_{disp}$*). So, by Theorem 3.B.16 in [2], it implies that* $X\leq_{dil\;}Y$.

The CRE and CE measures can be expressed in terms of the functions ${\bar{A}}_{X}\left(p\right)$ and $A_{X}\left(p\right)$, respectively. Recalling (7), and applying integration by parts with u=−log(1−p) and $dv=\left[F^{-1}\left(p\right)-E\left(X\right)\right]dp$, and thus $du=\frac{1}{1-p}dp$ and $v=-{\bar{A}}_{X}\left(p\right)$, we obtain an alternative expression for the CRE in terms of ${\bar{A}}_{X}\left(p\right)$ as follows:

(9) $$\begin{array}{ll} CRE\left(X\right)= & -\int_{0}^{1}\left[F^{-1}\left(p\right)-E\left(X\right)\right]\;\log\left(1-p\right)dp\\ & =\int_{0}^{1}\;\;\frac{1}{1-p}{\bar{A}}_{X}\left(p\right)dp. \end{array}$$

Similarly, recalling (8), and applying integration by parts with u=log(p) and $dv=\left[F^{-1}\left(p\right)-E\left(X\right)\right]dp$, and thus $du=\frac{1}{p}dp$ and $v=A_{X}\left(p\right)$, we have an alternative expression in terms of $A_{X}\left(p\right)$ for the CE as follows:

(10) $$\begin{array}{ll} CE\left(X\right)= & \int_{0}^{1}\left[F^{-1}\left(p\right)-E\left(X\right)\right]\;\log\left(p\right)dp\\ & =-\int_{0}^{1}\;\;\frac{1}{p}A_{X}\left(p\right)dp. \end{array}$$

The following counterexample demonstrates that the converse of Theorem 1 is not necessarily true, that is, CRE(X)≤CRE(Y) to (CE(X)≤CE(Y)) does not imply $X\leq_{\mathrm{dil}\;}Y$.

**Example** **2.** *Let* X *and* Y *be random variables with cdfs* $F\left(x\right)=2x-x^{2},$ *and* $G\left(x\right)=x^{2},$ *for* 0<x<1*. It is straightforward to verify that* $CE\left(X\right)=0.187$ *and* $CE\left(Y\right)=0.222$ *which implies* $CE\left(X\right)<CE(Y)$*. Moreover, one can obtain*

$$A_{X}\left(p\right)=\frac{2}{3}\left(p-1+{\left(1-p\right)}^{\frac{3}{2}}\right),\;and\;A_{Y}\left(p\right)=\frac{2}{3}\left(p^{\frac{2}{3}}-p\right),$$

**Figure 1:**
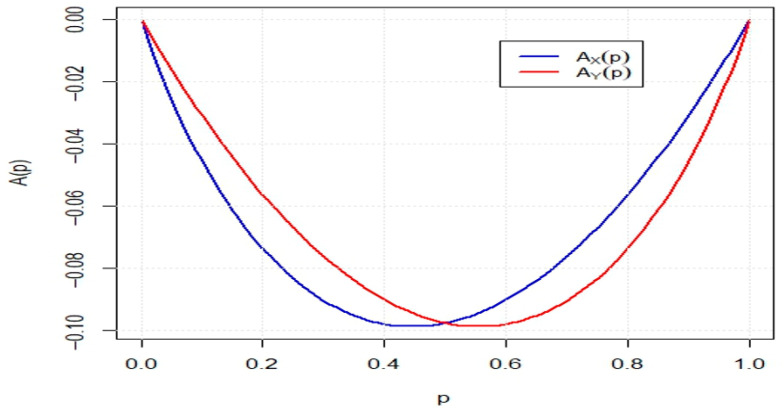
The plot of $A_{X}\left(p\right)$ and $A_{Y}\left(p\right)$ for all 0<p<1.

*for* 0<p<1. *Figure 1 displays the plots of* $A_{X}\left(p\right)$ *and* $A_{Y}\left(p\right)$ *over the interval* 0<p<1. *The figure reveals that* $A_{X}\left(p\right)<A_{Y}\left(p\right)$ *for* p∈(0,0.5) *and* $A_{X}\left(p\right)<A_{Y}\left(p\right)$ *for* p∈(0.5,1)*, leading to the conclusion that* $X{≰}_{dil}Y$.

The expressions presented in Equations (9) and (10) are now utilized to derive several important results. The following theorem emphasizes a key implication: if two random variables are ordered by dilation and possess the same CE, then they are identical in distribution or differ only by a location shift.

**Theorem** **2.** *Under the conditions of Theorem 1, if* $X\leq_{dil\;}Y$ *and* CE(X)=CE(Y)*, then* X *and* Y *have the same distribution up to a location parameter.*

**Proof.**  Based on the assumption CE(X)=CE(Y) and recalling (10), it is equivalent to

(11) $$\int_{0}^{1}\;\;\frac{1}{p}\left[A_{X}(p)-A_{Y}(p)\right]dp=0.$$

Furthermore, by Theorem 2.1 of [24], $X\leq_{\mathrm{dil}\;}Y$ implies

(12) $$A_{X}(p)\geq A_{Y}(p),0\leq p\leq 1.$$

From (11) and (12), $A_{X}(p)=A_{Y}(p)$ almost everywhere on [0,1]. We claim that $A_{X}(p)=A_{Y}(p)$ for all p∈[0,1]. Otherwise, there exists an interval (a,b)⊂[0,1] such that $A_{X}(p)>A_{Y}(p)$ for all p∈(a,b). Then,

$$\int_{0}^{1}\;\frac{1}{p}\left[A_{X}(p)-A_{Y}(p)\right]dp\geq \int_{a}^{b}\;\frac{1}{p}\left[A_{X}\left(p\right)-A_{Y}\left(p\right)\right]dp>0.$$

contradicting with (11). Therefore,

(13) $$\int_{p}^{1}\;\;\left[F^{-1}(t)-E(X)\right]dt=\int_{p}^{1}\;\;\left[G^{-1}(t)-E(Y)\right]dt,\;for\;all\;p\in [0,1].$$

Differentiating (13) with respect to p yields

$$F^{-1}\left(p\right)=G^{-1}\left(p\right)+E\left(X\right)-E\left(Y\right),\;\mathrm{for}\;\mathrm{all}\;p\in \left[0,1\right],$$

implying that X and Y have the same distribution up to a location parameter. □

For random variables ordered by dilation, equal CRE values imply identical distributions or differences only in location, which is proven in the next theorem.

**Theorem** **3.** *Under the conditions of Theorem 1, if* $X\leq_{dil\;}Y$ *and* CRE(X)=CRE(Y)*, then* X *and* Y *have the same distribution up to a location parameter.*

**Proof.**  Based on the assumption CRE(X)=CRE(Y) and recalling (9), it is equivalent to

(14) $$\int_{0}^{1}\;\;\frac{1}{1-p}\left[A_{X}(p)-A_{Y}(p)\right]dp=0.$$

Since ${\bar{A}}_{X}(p)=-A_{X}(p)$ for all 0≤p≤1, Theorem 2.1 of [24] implies that *X* ≤_dil_ *Y* leads to

(15) $${\bar{A}}_{X}(p)\leq {\bar{A}}_{Y}(p),0\leq p\leq 1$$

The remainder of the proof is analogous to that of Theorem 2. □

It should be noted that Theorems 2 and 3 imply that if $X\leq_{\mathrm{dil}\;}Y$ and CE(X)=CE(Y) (or CRE(X)=CRE(Y)), then $X\overset{\;\mathrm{dil}\;}{=}Y$, meaning X and Y are equal in distribution up to a location parameter.

## 3. Statistical Evidence for the Dilation Order via Cumulative Entropies

Economic and social processes often influence the variability of distributions, such as household spending before and after tax reforms, or stock returns before and after financial policy changes. A natural question in these contexts is whether such changes significantly alter variability. To address this, we develop tests for the null hypothesis $H_{0}:X\overset{\;\mathrm{dil}\;}{=}Y$ (variability remains unchanged) against the alternative $H_{1}:X\leq_{dil}Y$ and $X\overset{dil}{\neq }Y$ (variability increases). Note that $X\leq_{dil}Y$ and $Y\leq_{dil}X$ i.e., $X\overset{\;\mathrm{dil}\;}{=}Y$ if and only if $F\left(x\right)=G(x+d)$ for some real constant d and for all x. According to Theorems 1–3, this comparison can be expressed in terms of entropy-based evidence functions. In particular, the functionals

$$D_{1}(X,Y)=CRE\left(Y\right)-CRE\left(X\right)\;\mathrm{and}\;D_{2}(X,Y)=CE\left(Y\right)-CE\left(X\right),$$

serve as natural measures of departure from $H_{0}$ in favor of $H_{1}$. Thus, the null hypothesis should be rejected if the absolute values of $D_{1}(X,Y)$ or $D_{2}(X,Y)$ exceed their corresponding critical thresholds. Since the true values of $CRE\left(X\right)$, $CRE\left(Y\right)$, $CE\left(X\right)$, and $CE\left(Y\right)$ are generally unknown, we estimate them from independent random samples $\left\{X_{1},X_{2},\ldots ,X_{n}\;\right\}$ and $\left\{Y_{1},Y_{2},\ldots ,Y_{m}\;\right\}.$ Replacing the population entropies with their empirical counterparts, we obtain the test statistics

(16) $${\hat{D}}_{1}(n,m)={\hat{CRE}}_{m}(Y)-{\hat{CRE}}_{n}(X),$$

and

(17) $${\hat{D}}_{2}(n,m)={\hat{CE}}_{m}\left(Y\right)-{\hat{CE}}_{n}\left(X\right).$$

where ${\hat{CRE}}_{n}(.)$, ${\hat{CRE}}_{m}\left(.\right),$ ${\hat{CE}}_{n}\left(.\right)$ and ${\hat{CE}}_{m}\left(.\right)$ denote the empirical estimators of CRE and CE, for random samples from X and Y, respectively. Under the null hypothesis $X\overset{\;\mathrm{dil}\;}{=}Y$ i.e., the distributions of X and Y differ at most by a location shift; both population contrasts $D_{1}(X,Y)$ and $D_{2}(X,Y)$ are equal to zero (see Theorems 2 and 3). Large absolute values of the estimators ${\hat{D}}_{1}\left(n,m\right)$ and ${\hat{D}}_{2}\left(n,m\right)$ thus provide evidence against the null hypothesis. Importantly, while $X\leq_{dil}Y$ implies $D_{1}\left(X,Y\right)>0$ and $D_{2}\left(X,Y\right)>0$ (see Theorem 1), the converse does not hold (Example 2). Therefore, the sign of the estimated contrast should not be interpreted as definitive evidence for a specific direction of the dilation order. Instead, the statistics function as evidence functions, with the formal two-sided test providing protection against false claims of difference when $X\overset{\;\mathrm{dil}\;}{=}Y$. Thus, the null hypothesis should be rejected if $|{\hat{D}}_{1}\left(n,m\right)|>c_{1}$ and $|{\hat{D}}_{2}\left(n,m\right)|>c_{2}$. Here, the rejection thresholds $c_{1}$ and $c_{2}$ are determined by the null distributions of ${\hat{D}}_{1}(n,m)$ and ${\hat{D}}_{2}(n,m)$, which are studied in the next subsection. We reject $H_{0}$ when the estimates of ${\hat{D}}_{1}(n,m)$ or ${\hat{D}}_{2}(n,m)$ are sufficiently large. Let $X_{1},X_{2},\ldots ,X_{n}$ be a sequence of independent and identically distributed (i.i.d.) continuous nonnegative random variables, with order statistics $X_{1:n}\leq X_{2:n}\leq \ldots \leq X_{n:n}$. The empirical distribution function corresponding to F(x) is defined as

$${\hat{F}}_{n}\left(x\right)=\frac{1}{n}\sum_{i=1}^{n}\;I_{\left(X_{i}\leq x\right)},$$

which can equivalently be expressed as

$${\hat{F}}_{n}(x)=\left\{\begin{array}{l} 0,x<x_{1:n}\\ \frac{i}{n},x_{i:n}\leq x\leq x_{i+1:n},(i=0,1,2,\ldots ,n-1)\\ 1,x>x_{n:n} \end{array}\right.$$

where $I_{A}$ denotes the indicator function of event A. An estimator of the CRE, based on a nonparametric approach and derived from the *L*-functional estimator, is then given by

$${\hat{CRE}}_{n}\left(X\right)=\int_{0}^{\infty }\;xJ\left({\hat{F}}_{n}\left(x\right)\right)d{\hat{F}}_{n}\left(x\right)=\frac{1}{n}\sum_{i=0}^{n-1}\;J\left(\frac{i}{n}\right)X_{i:n},$$

where J(u)=−log(1−u)−1, for 0<u<1. Similar arguments can be applied to obtain the estimator ${\hat{CRE}}_{m}(Y)$. The following theorem demonstrates mean, variance and RMSE of ${\hat{CRE}}_{n}\left(X\right)$ is invariant to shifts in the random variable X, but not to scale transformations.

**Theorem** **4.** *Assume that* $X_{1},X_{2},\ldots ,X_{n}$ *is a random sample of size* n *taken from a population with pdf* f *and cdf* F *and. Then, when* n *is approximately large, the following properties apply:*

*(i)* 
$E\left({\hat{CRE}}_{n}\left(aX+b\right)\right)=aE\left({\hat{CRE}}_{n}\left(X\right)\right)$ *,*
*(ii)* 
$Var\left({\hat{CRE}}_{n}\left(aX+b\right)\right)=a^{2}Var\left({\hat{CRE}}_{n}\left(X\right)\right)$ *,*
*(iii)*  $RMSE\left({\hat{CRE}}_{n}\left(aX+b\right)\right)=aRMSE\left({\hat{CRE}}_{n}\left(X\right)\right)$.

**Proof.**  It is not hard to see that

$$\begin{array}{ll} {\hat{CRE}}_{n}\left(aX+b\right) & =\frac{1}{n}\sum_{i=0}^{n-1}\;J\left(\frac{i}{n}\right){(aX}_{i:n}+b)\\ & =\frac{a}{n}\sum_{i=0}^{n-1}\;J\left(\frac{i}{n}\right)X_{i:n}+\frac{b}{n}\sum_{i=0}^{n-1}\;J\left(\frac{i}{n}\right)\\ & =a{\hat{CRE}}_{n}\left(X\right). \end{array}$$

The last equality is obtained by noting that

$$\frac{1}{n}\sum_{i=0}^{n-1}\;J\left(\frac{i}{n}\right)=\frac{1}{n}\sum_{i=0}^{n-1}\;\left(-\log\left(\frac{n-i}{n}\right)-1\right)\approx 0,$$

since

$$\frac{1}{n}\sum_{i=0}^{n-1}\;\left(-\log\left(\frac{n-i}{n}\right)-1\right)$$

is a Riemann sum for the integral of $\int_{0}^{1}\left(-\log\left(1-x\right)-1\right)dx=0,$ when n is approximately large. The proof is then completed by leveraging the properties of the mean, variance, and RMSE of ${\hat{CRE}}_{n}\left(aX+b\right)=a{\hat{CRE}}_{n}\left(X\right)$. □

Similar arguments can be applied to obtain the estimator ${\hat{CRE}}_{m}(Y)$. Since ${\hat{D}}_{1}(n,m)$ is a linear combination of the dispersion measures ${\hat{CRE}}_{n}\left(X\right)$ and ${\hat{CRE}}_{m}(Y)$, the results established in Theorem 4 for ${\hat{D}}_{1}(n,m)$ follow directly from the corresponding properties of these estimators. The following theorem establishes the asymptotic normality of the test statistic ${\hat{D}}_{1}(n,m)$, providing the theoretical foundation for its use as an evidence function in testing the dilation order.

**Theorem** **5.** *Assume that* $E\left(X^{2}\right)<\infty$ *and* $E\left(Y^{2}\right)<\infty$ *with* $\sigma^{2}(F)>0$*, and* $\sigma^{2}(G)>0$*. Let* S=m+n *and suppose that for some* 0<τ<1*,* $\frac{n}{S}\to \tau ,\;\frac{m}{S}\to 1-\tau \;\;\mathrm{as}\;min\left\{n,m\right\}\to \infty .$ *Then, as* min{n,m}→∞*,* $\sqrt{S}\left({\hat{D}}_{1}(n,m)-D_{1}(X,Y)\right)\to_{d}N\left(0,\sigma^{2}(F,G)\right)$*, where*

$$\sigma^{2}(F,G)=\frac{\sigma^{2}(F)}{\tau }+\frac{\sigma^{2}(G)}{1-\tau },$$

*with*

(18) $$\sigma^{2}\left(F\right)=\int_{0}^{\infty }\;\;\int_{0}^{\infty }\;\;\left(F\left(min\left(x,y\right)\right)-F\left(x\right)F\left(y\right)\right)J\left(x\right)J\left(y\right)dx\;dy,$$

*and* $\sigma^{2}(G)$ *defined analogously.*

**Proof.**  Since the function J is bounded and continuous, Theorems 2 and 3 of [30] imply that $\sqrt{n}\left({\hat{CRE}}_{n}(X)-CRE(X)\right)$ converges in distribution to a normal law with mean zero and finite variance $\sigma^{2}(F)>0$ as n→∞. A similar convergence holds for ${\hat{E}}_{m}(Y)$, because convergence in distribution is preserved under convolution.

To address the dependence on the unknown distribution function, we employ a consistent estimator of the variance. Following the representation of [31], we define

$${\hat{\sigma }}_{n}^{2}(F)=\sum_{i=0}^{n-1}\;\sum_{j=0}^{n-1}\;\left(min\left(\frac{i}{n},\frac{j}{n}\right)-\frac{i}{n}\frac{j}{n}\right)J\left(\frac{i}{n}\right)J\left(\frac{j}{n}\right)\left(X_{i+1:n}-X_{i:n}\right)\left(X_{j+1:n}-X_{j:n}\right),$$

with ${\hat{\sigma }}_{m}^{2}(G)$ defined analogously. The decision rule for rejecting $H_{0}$ in favor of $H_{1}$ at significance level α is:

(19) $$\left|\frac{{\hat{D}}_{1}(n,m)}{\sqrt{\frac{{\hat{\sigma }}_{n}^{2}(F)}{n}+\frac{{\hat{\sigma }}_{m}^{2}(G)}{m}}}\right|>z_{1-\frac{q}{2}},$$

where $z_{1-\frac{q}{2}}$ represents the $\left(1-\frac{q}{2}\right)$-quantile of the standard normal distribution. □

We now present the analogous result for the cumulative entropy. To this end, we propose a nonparametric estimator of CE, derived from the L-functional representation, defined as:

$${\hat{CE}}_{n}\left(X\right)=\int_{0}^{\infty }\;x\bar{J}\left({\hat{F}}_{n}\left(x\right)\right)d{\hat{F}}_{n}\left(x\right)=\frac{1}{n}\sum_{i=0}^{n-1}\;\bar{J}\left(\frac{i}{n}\right)X_{i:n},$$

where $\bar{J}(u)=log(u)+1$, for 0<u<1. A similar estimator can be constructed for ${\hat{CE}}_{m}(Y)$. Similar Theorem 4 can also be obtained for the estimator ${\hat{CE}}_{n}\left(X\right)$.

**Theorem** **6.** *Assume that* $X_{1},X_{2},\ldots ,X_{n}$ *is a random sample of size* n *taken from a population with pdf* f *and cdf* F *and. Then, when* n *is approximately large, the following properties apply:*

*(i)* 
$E\left({\hat{CE}}_{n}\left(aX+b\right)\right)=aE\left({\hat{CE}}_{n}\left(X\right)\right)$ *,*
*(ii)* 
$Var\left({\hat{CE}}_{n}\left(aX+b\right)\right)=a^{2}Var\left({\hat{CE}}_{n}\left(X\right)\right)$ *,*
*(iii)*  $RMSE\left({\hat{CE}}_{n}\left(aX+b\right)\right)=aRMSE\left({\hat{CE}}_{n}\left(X\right)\right)$.

Similar arguments can be applied to obtain the estimator ${\hat{CE}}_{m}(Y)$. Since ${\hat{D}}_{2}(n,m)$ is a linear combination of the dispersion measures ${\hat{CE}}_{n}\left(X\right)$ and ${\hat{CE}}_{m}(Y)$, the results established in Theorem 6 for ${\hat{D}}_{2}(n,m)$ follow directly from the corresponding properties of these estimators. The asymptotic normality of the CE-based test statistic ${\hat{D}}_{2}(n,m)$ is established in the following theorem. Since its proof closely parallels that of Theorem 4, it is omitted here for brevity.

**Theorem** **7.** *Assume that* $E\left(X^{2}\right)<\infty$ *and* $E\left(Y^{2}\right)<\infty$ *such that* ${\bar{\sigma }}^{2}(F)>0$*, and* ${\bar{\sigma }}^{2}(G)>0$*. Let* S=m+n *and suppose that for some* 0<τ<1*, we have*

$$\frac{n}{S}\to \tau ,\frac{m}{S}\to 1-\tau \;\mathrm{as}\;min\left\{n,m\right\}\to \infty .$$

*Then, as* min{n,m}→∞*,* $\sqrt{S}\left({\hat{D}}_{2}(n,m)-D_{2}(X,Y)\right)$ *is normal with mean zero and the finite variance*

$${\bar{\sigma }}_{n,m}^{2}(F,G)=\frac{{\bar{\sigma }}_{n}^{2}(F)}{\tau }+\frac{{\bar{\sigma }}_{m}^{2}(G)}{1-\tau },$$

*where*

(20) $${\bar{\sigma }}^{2}\left(F\right)=\int_{0}^{\infty }\;\;\int_{0}^{\infty }\;\;\left(F\left(min\left(x,y\right)\right)-F\left(x\right)F\left(y\right)\right)\bar{J}\left(x\right)\bar{J}\left(y\right)dx\;dy,$$

*and* ${\bar{\sigma }}^{2}(G)$ *defined analogously.*

*The estimator for* ${\bar{\sigma }}^{2}(F)$ *is defined as:*

$${\hat{\bar{\sigma }}}_{n}^{2}(F)=\sum_{i=1}^{n-1}\;\sum_{j=1}^{n-1}\;\left(min\left(\frac{i}{n},\frac{j}{n}\right)-\frac{i}{n}\frac{j}{n}\right)\bar{J}\left(\frac{i}{n}\right)\bar{J}\left(\frac{j}{n}\right)\left(X_{i+1:n}-X_{i:n}\right)\left(X_{j+1:n}-X_{j:n}\right).$$

*Similarly, we estimate* ${\bar{\sigma }}^{2}(G)$ *as* ${\hat{\bar{\sigma }}}_{m}^{2}(G)$*. Consequently, the decision rule for rejecting* $H_{0}$ *in favor of* $H_{1}$ *at significance level q is:*

(21) $$\left|\frac{{\hat{D}}_{2}(n,m)}{\sqrt{\frac{{\hat{\bar{\sigma }}}_{n}^{2}(F)}{n}+\frac{{\hat{\bar{\sigma }}}_{m}^{2}(G)}{m}}}\right|>z_{1-\frac{q}{2}},$$

*where* $z_{1-\frac{q}{2}}$ *is defined previously.*

**Remark** **2.** *An important feature of the dilation order is that it provides a natural framework for characterizing the harmonic new better than used in expectation (HNBUE) and harmonic new worse than used in expectation (HNWUE) aging classes [32,33]. Specifically, a random variable* X *belongs to the HNBUE (respectively, HNWUE) class if and only if* $X\leq_{dil}Y$ *for some* Y *that is exponential with mean equal to that of* X*, i.e.,* E(Y)=E(X)*. Building on this foundational concept, we introduce a test statistic that can be employed to evaluate the null hypothesis:* $H_{0}^{\prime }$*:* X *follows an exponential distribution vs.* $H_{1}^{\prime }:X$ *belongs to the HNBUE or HNWUE class but does not conform to an exponential distribution.*

*If* Y *represents a random variable with an exponential distribution with mean of* E(X)*, we can derive*

$${D^{\prime }}_{1}(X,Y)=2E\left(X\right)-\int_{0}^{\infty }\;x\log\left(1-F\left(x\right)\right)dF\left(x\right),$$

*and*

$${D^{\prime }}_{2}(X,Y)=-\int_{0}^{\infty }\;x\log F\left(x\right)dF\left(x\right)-E\left(X\right)\left(\frac{\pi^{2}}{16}-2\right),$$

*where measures quantify deviation from* $H_{0}^{\prime }$ *to* $H_{1}^{\prime }$*. The measures are empirically estimated, respectively, as:*

$${\hat{D}\prime }_{1}(n,m)=\frac{1}{n}\sum_{i=0}^{n-1}\;J^{\prime }\left(\frac{i}{n}\right)X_{i:n},\;\;\mathrm{and}\;\;{\hat{D^{\prime }}}_{2}(n,m)=\frac{1}{n}\sum_{i=0}^{n-1}\;{\bar{J}}^{\prime }\left(\frac{i}{n}\right)X_{i:n},$$

*where* $J^{\prime }\left(u\right)=2-log(1-u)$ *and* ${\bar{J}}^{\prime }(u)=-log(u)-\left(\frac{\pi^{2}}{16}-2\right)$ *for* 0<u<1. *Similar to Theorem 4 can also be obtained for the estimators* ${\hat{D}\prime }_{1}(n,m)$ *and* ${\hat{D}\prime }_{2}(n,m)$*. To obtain scale invariant tests, we can use the statistics* ${\hat{D}}_{1}^{HNBUE}(n,m)={\hat{D^{\prime }}}_{1}(n,m)/\bar{X}$ *and* ${\hat{D}}_{2}^{HNBUE}(n,m)={\hat{D^{\prime }}}_{2}(n,m)/\bar{X}$*, where* $\bar{X}$ *represents the sample mean. By similar arguments as in the proofs of Theorems 5 and 7, and applying Slutsky’s theorem, we obtain the following asymptotic distributions:*

$$\sqrt{n}\left({\hat{D}}_{1}^{HNBUE}(n,m)-\frac{{D^{\prime }}_{1}(X,Y)}{E(X)}\right)\overset{d\;}{\to }N\left(0,\frac{\sigma_{J^{\prime }}^{2}(F)}{E^{2}(X)}\right),$$

*and*

$$\sqrt{n}\left({\hat{D}}_{2}^{HNBUE}(n,m)-\frac{{D^{\prime }}_{2}(X,Y)}{E(X)}\right)\overset{d\;}{\to }N\left(0,\frac{\sigma_{{\bar{J}}^{\prime }}^{2}(F)}{E^{2}(X)}\right).$$

*The null hypothesis* $H_{0}^{\prime }$ *should be rejected if*

$$\left|\sqrt{\frac{n}{{\hat{\sigma }}_{n,J^{\prime }}^{2}(F)}}{\hat{D^{\prime }}}_{1}(n,m)\right|>z_{1-\frac{q}{2}},\;\;\mathrm{and}\;\left|\sqrt{\frac{n}{{\hat{\sigma }}_{n,{\bar{J}}^{\prime }}^{2}(F)}}{\hat{D^{\prime }}}_{2}(n,m)\right|>z_{1-\frac{q}{2}},$$

*where* ${\hat{\sigma }}_{n,J^{\prime }}^{2}(F)$ *and* ${\hat{\sigma }}_{n,{\bar{J}}^{\prime }}^{2}(F)$ *are the estimators of* $\sigma_{J^{\prime }}^{2}(F)$ *and* $\sigma_{{\bar{J}}^{,}}^{2}(F)$*, respectively.*

### 3.1. Simulation Study

To assess the finite-sample performance of the proposed tests in (19) and (21), we carried out a simulation study comparing their power functions across a range of representative probability models. The chosen distributions are widely applied in economics, finance, insurance, and reliability, and together they span scenarios from light-tailed to heavy-tailed behavior. As a natural benchmark, we first considered the exponential distribution, a standard reference model in reliability theory whose tail behavior provides a baseline for detecting departures toward heavier-tailed alternatives. To capture such heavy-tailed phenomena, we included the Pareto distribution, commonly employed in economics, finance, and insurance to model extreme events. Its scale and shape parameters strongly affect dispersion, making it particularly relevant for testing under the dilation order. We also examined the gamma distribution, a versatile model frequently used in econometrics, Bayesian analysis, and life-testing. Its shape–scale parameterization offers flexibility in modeling waiting times, and in the special case of integer shape parameters it reduces to the Erlang distribution. Finally, we incorporated the Weibull distribution, another classical lifetime model with broad applications in reliability and survival analysis, well known for its ability to describe diverse aging behaviors. Together, these four families, exponential, Pareto, gamma, and Weibull, provide a balanced experimental design that reflects both exponential-tail and long-tail settings. For comparability and to ensure meaningful stochastic orderings, all simulated distributions were standardized to share a common mean, although this constraint is not required for the theoretical validity of the proposed tests. We evaluated our proposed tests by comparing their empirical power against four recent tests for the dilation order. Specifically, we compared our statistics ${\hat{D}}_{1}(n,m)$ and ${\hat{D}}_{2}(n,m)$ to those developed by the following test statistics:

- Aly’s $t_{N}$ statistic [28]:
  $$t_{N}=\delta_{m}\left(G\right)-\delta_{n}\left(F\right),$$
  where
  $$\delta_{m}(G)=\frac{1}{m^{2}}\sum_{i=2}^{m}\;(i-1)(m-i+1)\left(X_{i:m}-X_{i-1:m}\right),$$
  and $\delta_{n}(F)$ is defined similarly.
- The test statistic ${\hat{\Delta }}_{dil}^{\alpha_{1}}(X,Y)$ proposed by Belzunce et al. [32]:
  $${\hat{\Delta }}_{dil}^{\alpha_{1}}(X,Y)=\frac{1}{n}\sum_{i=1}^{n}\;\left(c_{i,n}^{\alpha_{1}}-\frac{1-\alpha_{1}^{2}}{6}\right)X_{i:n}-\frac{1}{m}\sum_{i=1}^{m}\;\left(c_{i,m}^{\alpha_{1}}-\frac{1-\alpha_{1}^{2}}{6}\right)Y_{i:n},$$
  where
  $$c_{i,r}^{\alpha_{1}}=\left\{\begin{array}{ll} \left(\frac{1}{\alpha_{1}}-1\right)\frac{3r^{2}\alpha_{1}-3i^{2}+3i-1}{6r^{2}} & \;\mathrm{if}\;i<k+1\\ \frac{3\alpha_{1}(r-k-1{)}^{2}+(k-r\alpha_{1}{)}^{3}+\alpha_{1}(3r-3k-2)}{6r^{2}\alpha_{1}} & \;\mathrm{if}\;i=k+1\\ \frac{3(r-i{)}^{2}+3(r-i)+1}{6r^{2}} & \;\mathrm{if}\;i>k+1, \end{array}\right.$$
  with $\frac{k}{r}\leq \alpha_{1}<\frac{k+1}{r}$.
- The test statistic $\hat{\Delta }(n,m)$ introduced by [33], which is based on Gini’s mean difference:
  $$\hat{\Delta }\left(n,m\right)=\frac{m+1}{m\left(m-1\right)}\sum_{i=1}^{m}\;2\left(\frac{2i}{m+1}-1\right)Y_{i:m}-\frac{n+1}{n\left(n-1\right)}\sum_{i=1}^{n}\;2\left(\frac{2i}{n+1}-1\right)X_{i:n}.$$
- Zardasht’s $T_{nm}^{\alpha_{2}}$ statistic [29], defined as:
  $$T_{nm}^{\alpha_{2}}=\frac{1}{m}\sum_{i=1}^{m}\;J_{\alpha_{2}}\left(\frac{i}{m}\right)Y_{i:m}-\frac{1}{n}\sum_{i=1}^{n}\;J_{\alpha_{2}}\left(\frac{i}{n}\right)X_{i:n},$$
  where $J_{\alpha_{2}}(u)=\frac{1}{1-\alpha_{2}}\left[1-\alpha_{2}(1-u{)}^{\alpha_{2}-1}\right]$ for all $\alpha_{2}>0$ and $\alpha_{2}\neq 1$.

The statistic ${\hat{\Delta }}_{dil}^{\alpha_{1}}(X,Y)$ from [32] depends on a parameter $\alpha_{1}\in (0,1)$; since its performance remains largely consistent across different $\alpha_{1}$ values, we adopted $\alpha_{1}=0.5$ for our analysis. Similarly, for $T_{nm}^{\alpha_{2}}$ from [29], we chose $\alpha_{2}=2$. We also simulated the following scenarios which are tabulated in Table 1 and compared the empirical powers of the test statistics.

(i) *Exponential Distribution*: For this scenario, X∼E(1) and Y∼E(1/β) where β is varied from 1 to 2. The null hypothesis is then represented by the case where β=1.
(ii) *Pareto Distribution*: For this scenario, the random variable X∼P(10,3) and Y∼P(10/β,3) where β varied from 1 to 2. The null hypothesis is then represented by the case where β=1.
(iii) *Gamma Distribution*: For this scenario, X∼G(2,1) and Y∼G(β,1) where β varied from 2 to 3. The null hypothesis is then represented by the case where β=2.
(iv) *Weibull Distribution*: For this scenario, X∼W(2,1) and Y∼W(β,1) where β varied from 1 to 2. The null hypothesis is then represented by the case where β=2.
(v) *Mixture Weibull Distribution*: For this scenario, we have
  $$f_{Z}\left(z\right)=0.5[f_{X}\left(z\right)+f_{Y}\left(z\right)]\;z>0,$$
  where X∼W(2,1) and Y∼W(β,1) where β varied from 1 to 2. The null hypothesis is then represented by the case where β=2.

**Figure 2:**
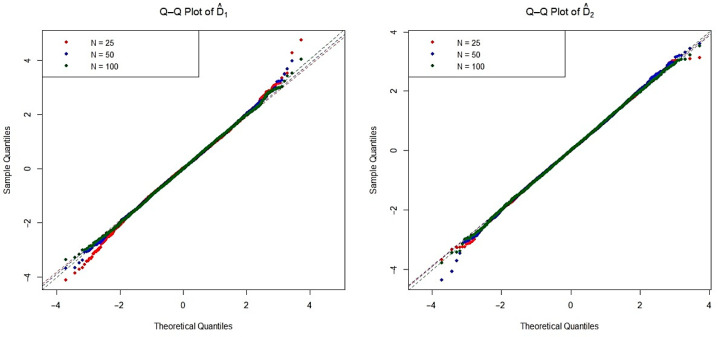
The Q-Q plots of the estimators ${\hat{D}}_{1}(n,m)$ and ${\hat{D}}_{2}(n,m)$ obtained from simulations under the exponential distribution for various sample sizes.

**Figure 3:**
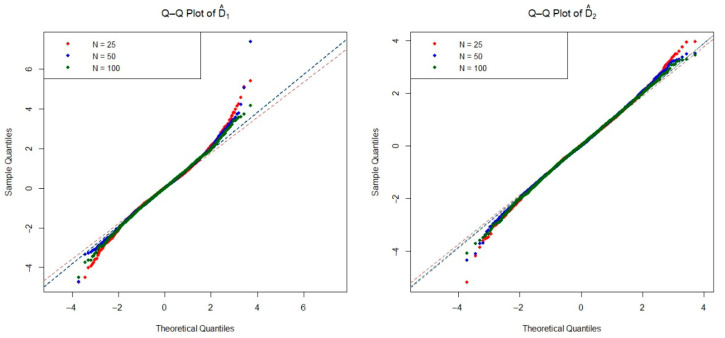
The Q-Q plots of the estimators ${\hat{D}}_{1}(n,m)$ and ${\hat{D}}_{2}(n,m)$ obtained from simulations under the Pareto distribution for various sample sizes.

**Figure 4:**
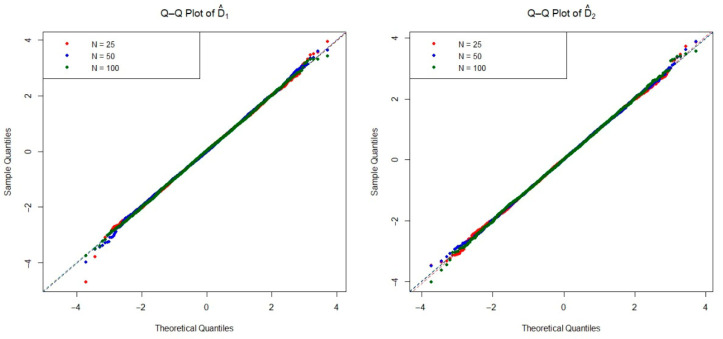
The Q-Q plots of the estimators ${\hat{D}}_{1}(n,m)$ and ${\hat{D}}_{2}(n,m)$ obtained from simulations under the Weibull distribution for various sample sizes.

**Figure 5:**
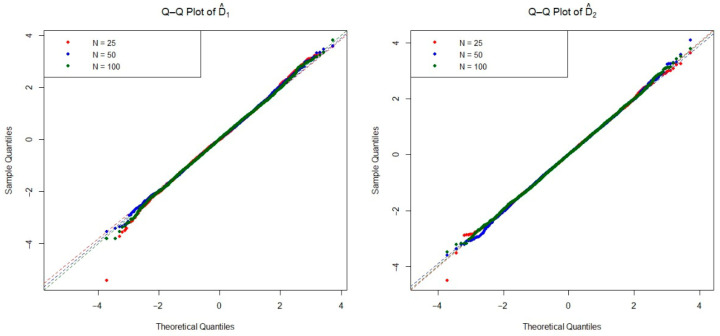
The Q-Q plots of the estimators ${\hat{D}}_{1}(n,m)$ and ${\hat{D}}_{2}(n,m)$ obtained from simulations under the gamma distribution for various sample sizes.

**Figure 6:**
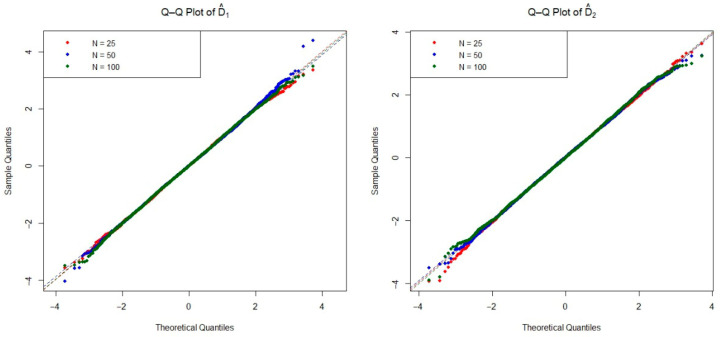
The Q-Q plots of the estimators ${\hat{D}}_{1}(n,m)$ and ${\hat{D}}_{2}(n,m)$ obtained from simulations under the mixture Weibull distribution for various sample sizes.

The asymptotic distribution of the test statistic is crucial for determining the critical values used to decide on the null hypothesis. To examine the empirical densities of the estimators ${\hat{D}}_{1}(n,m)$ and ${\hat{D}}_{2}(n,m)$ under the null hypothesis, we conducted an extensive simulation study based on the Monte Carlo method. Specifically, we generated 20,000 independent iterations pairs of random samples from each of the four distributions listed in Table 1, as well as the mixture Weibull distribution. For each distribution, we considered three sample size configurations n=m=25, 50, and 100 under the null hypothesis. The Q-Q plots of two estimators are presented in Figure 2, Figure 3, Figure 4, Figure 5 and Figure 6. These plots provide valuable insight into the shape, center, and spread of the estimators’ distributions under $H_{0}$ and illustrate the convergence toward normality as the sample size increases, consistent with asymptotic theory.

To obtain the critical values of estimators ${\hat{D}}_{1}(n,m)$ and ${\hat{D}}_{2}(n,m)$, we iterate 5000 samples of size n=m from the null hypothesis distributions. From the 5000 values of estimators ${\hat{D}}_{1}(n,m)$ and ${\hat{D}}_{2}(n,m)$, (1 − q)th quantile represents the critical value corresponding to sample size n=m of the test statistics at significance level q. If the critical values for the two-sided test are denoted as ${\hat{D}}_{1,1-\frac{q}{2}}(n,m)$ and ${\hat{D}}_{2,1-\frac{q}{2}}(n,m)$, then the null hypothesis is rejected with size q whenever $\left|{\hat{D}}_{1}\left(n,m\right)|>{\hat{D}}_{1,1-\frac{q}{2}}(n,m\right)$ and $\left|{{\hat{D}}_{1}\left(n,m\right)|>\hat{D}}_{2,1-\frac{q}{2}}(n,m\right)$. The critical values of ${\hat{D}}_{1,1-\frac{q}{2}}(n,m)$ and ${\hat{D}}_{2,1-\frac{q}{2}}(n,m)$ are based on 5000 samples of different sample sizes generated from the null distribution at significance level q= 0.05. The empirical power of the proposed test statistics was evaluated via five distribution functions, exponential, Pareto, gamma, Weibull, and mixture Weibull, using 5000 independent pairs of random samples for each configuration, with equal sample sizes n=m=25, 50, and 100. For each replication, the null hypothesis of dilation equivalence was tested, and empirical power was calculated as the proportion of rejections among the 5000 simulations. The results, summarized in Table 2, Table 3, Table 4, Table 5 and Table 6, confirm the expected consistency: power generally increases with sample size for all methods.

Notably, the CE-based statistic ${\hat{D}}_{2}(n,m)$ exhibits superior power in most scenarios, particularly for exponential, Pareto, and gamma distributions. This underscores its effectiveness in detecting deviations from exponentiality toward heavier-tailed or more dispersed alternatives within these families. However, ${\hat{D}}_{2}(n,m)$ shows weaker performance under Weibull and mixture Weibull alternatives, indicating limited sensitivity to the specific dispersion characteristics of this model. In contrast, the CRE-based statistic ${\hat{D}}_{1}(n,m)$ demonstrates modest power in small samples but markedly improves as sample size grows, especially in Weibull and mixture Weibull settings, where it often outperforms competing methods including ${\hat{D}}_{2}(n,m)$ at n=m=100. This suggests that ${\hat{D}}_{1}(n,m)$ gains reliability and robustness with larger datasets for these distributions.

Overall, while ${\hat{D}}_{2}(n,m)$ emerges as a versatile and powerful tool across a broad range of distributions, its suitability depends on the underlying data-generating process. Likewise, ${\hat{D}}_{1}(n,m)$ offers complementary strengths, particularly in Weibull-type or heavy-tailed mixture contexts. Thus, the two statistics are best viewed as complementary evidence functions, with the choice between them guided by the nature of the distribution and the available sample size.

### 3.2. Real-Data Analysis

To illustrate the practical utility of the proposed methodology, we analyze a real dataset on survival times of male RFM strain mice, originally reported by [34]. The study considered two groups: the first group (X) consisted of mice raised under conventional laboratory conditions, while the second group (Y) was raised in a germ-free environment. In both groups, death was due to thymic lymphoma, allowing a direct comparison of survival variability under different environmental conditions. This dataset is particularly valuable in survival and reliability analysis because it allows us to investigate how external factors, in this case environmental exposure, affect the dispersion of lifetimes. Specifically, our interest lies in testing whether the survival distribution of mice raised in germ-free conditions (Y) is more dispersed than that of mice raised conventionally (X), which corresponds to verifying the dilation order relationship $X\leq_{dil}Y$.

As a preliminary step, we followed the graphical approach recommended by [5], which suggested evidence consistent with the dilation ordering $X\leq_{dil}Y$. Building on this, we applied six test statistics, including the proposed entropy-based measures ${\hat{D}}_{1}(n,m)$ and ${\hat{D}}_{2}(n,m)$, to formally assess the hypothesis. The results, summarized in Table 7, provide strong statistical support for the dilation order. In particular, all six test statistics yielded small *p*-values, leading to rejection of the null hypothesis of equality and confirming the alternative $X\leq_{dil}Y$ in significance level q=0.05. The entropy-based test ${\hat{D}}_{2}$ was especially effective, delivering the strongest evidence among the six statistics.

This real-data application highlights three key insights. First, it demonstrates how cumulative entropies can function as practical evidence measures, capable of validating stochastic orderings in empirical settings. Second, it shows that entropy-based tests can reveal differences in variability between populations that may not be apparent from mean comparisons alone. Third, it illustrates the robustness and versatility of the proposed methodology in survival data analysis, with implications extending to biomedical research, reliability engineering, and actuarial science. This validation on real data underscores that entropy-based evidence functions are not only theoretically sound but also practically reliable, even in complex biological survival settings.

## 4. Conclusions

This paper introduced novel classes of entropy-based test statistics for assessing the dilation order, one rooted in an evidential interpretation of stochastic variability. By leveraging CRE and CE, we constructed evidence functions that quantify the degree to which one distribution exhibits greater variability than another, without requiring parametric assumptions. These statistics not only offered a principled measure of divergence aligned with dilation ordering but also served as interpretable evidence metrics in hypothesis testing. Moreover, we established the theoretical foundation of the proposed methods by deriving their asymptotic distributions and analyzing their large-sample behavior, ensuring validity under standard regularity conditions. Extensive simulation studies demonstrated that the CE-based statistic achieves high power and strong consistency across diverse alternatives, even in moderate samples, while the CRE-based counterpart exhibited notable robustness and improved performance as sample size increases. This complementary behavior underscored their joint utility across both small- and large-sample regimes. The practical relevance of the framework is illustrated through an analysis of survival times from RFM strain mice, where the proposed tests provide statistically significant evidence of dilation ordering between treatment groups. This real-world application highlights the methodology’s potential in survival analysis, reliability engineering, and biomedical research, where assessing variability differences is often more informative than mean comparisons.

On the other hand, the approach is extended to the classical problem of testing exponentiality against HNBUE and HNWUE alternatives, a critical task in reliability and actuarial science. The resulting tests inherited the evidential structure of the main framework, thereby bridging stochastic orders, aging properties, and information-theoretic measures in a unified setting. Despite these advances, several promising directions remain open for future work:

1. Refined inference procedures, such as bootstrap or permutation-based methods, to enhance small-sample accuracy in strength of evidence uncertainty estimation control of test Type I error.
2. Extension to multivariate settings, where notions of dilation order and entropy must be generalized to account for dependence and dimensionality.
3. Adaptation to time-dependent and censored data, including survival models with covariates, recurrent events, or temporal dependence structures (e.g., Markov or stationary processes).
4. Robustness analysis under model misspecification and integration into Bayesian evidence frameworks, potentially via entropy-based Bayes factors.
5. Generalization to alternative entropy measures, such as Rényi or Tsallis entropies, which may yield more flexible evidence functions adaptable to heavy-tailed or asymmetric distributions.

Together, these future avenues promise to broaden the scope, rigor, and applicability of entropy-based evidential testing in both theoretical and applied statistics.

## Figures and Tables

**Table 1:** Probability distributions with the shape parameter *β* and the scale parameter *γ*.

Distribution	Probability Density Function	Support
Exponential	$f(x)=\frac{1}{\gamma }e^{-\frac{x}{\gamma }}$,	x>0,γ>0
Pareto	$f(x)=\frac{\beta \gamma^{\beta }}{(x+\gamma {)}^{\beta +1}}$,	x>0,γ,β>0
Gamma	$f(x)=\frac{1}{\gamma^{\beta }\Gamma (\beta )}x^{\beta -1}e^{-\frac{x}{\gamma }}$	x>0,γ,β>0
Weibull	$f(x)=\frac{\beta }{\gamma^{\beta }}x^{\beta -1}e^{-{\left(\frac{x}{\gamma }\right)}^{\beta }}$,	x>0,γ,β>0
Mixture Weibull	$f\left(x\right)=0.5\left[2xe^{-x^{2}}+\beta x^{\beta -1}e^{-x^{\beta }}\right]$	x>0,β>0

**Table 2:** Power comparisons of the tests in significance level *q* = 0.05.

Exponential							
n=m	γ	$t_{N}$	${\hat{\Delta }}_{\mathrm{dil}\;}^{0.5}(X,Y)$	$\hat{\Delta }(n,m)$	$T_{nm}^{2}$	${\hat{D}}_{1}(n,m)$	${\hat{D}}_{2}(n,m)$
25	0.5	0.7176	0.7566	0.7444	0.7438	0.6622	0.8048
	0.6	0.4698	0.5096	0.4948	0.4966	0.4384	0.5470
	0.7	0.2746	0.3048	0.2860	0.2936	0.2544	0.3176
	0.8	0.1246	0.1506	0.1562	0.1524	0.1388	0.1662
	0.9	0.0732	0.0734	0.0734	0.0832	0.08	0.0808
	1.0	0.0418	0.0428	0.0558	0.0526	0.0614	0.0518
50	0.5	0.9288	0.9404	0.9380	0.9388	0.8642	0.9634
	0.6	0.7316	0.7392	0.7410	0.7326	0.6360	0.8022
	0.7	0.4412	0.4416	0.4480	0.4462	0.3584	0.5024
	0.8	0.2124	0.2066	0.2190	0.2156	0.1700	0.2440
	0.9	0.0864	0.0886	0.0938	0.0888	0.0844	0.0936
	1.0	0.0498	0.0490	0.0512	0.0526	0.0500	0.0488
100	0.5	0.9962	0.9976	0.9970	0.9962	0.9840	0.9988
	0.6	0.9336	0.9444	0.9454	0.9336	0.8730	0.9646
	0.7	0.6846	0.7012	0.7004	0.6856	0.5878	0.7498
	0.8	0.3164	0.3324	0.3446	0.3356	0.2872	0.3946
	0.9	0.1042	0.1050	0.1228	0.1014	0.1100	0.1432
	1.0	0.0406	0.0462	0.0454	0.0442	0.0518	0.0526

**Table 3:** Power comparisons of the tests in significance level *q* = 0.05.

Pareto							
n=m	β	$t_{N}$	${\hat{\Delta }}_{\mathrm{dil}\;}^{0.5}(X,Y)$	$\hat{\Delta }(n,m)$	$T_{nm}^{2}$	${\hat{D}}_{1}(n,m)$	${\hat{D}}_{2}(n,m)$
25	1.0	0.0510	0.0454	0.0496	0.0556	0.0486	0.0398
	1.2	0.1276	0.1100	0.1176	0.1254	0.1024	0.1176
	1.4	0.2654	0.2704	0.2656	0.2914	0.2156	0.2748
	1.6	0.4420	0.4602	0.4460	0.4876	0.3772	0.4926
	1.8	0.6454	0.6472	0.6250	0.6536	0.5460	0.6598
	2.0	0.7508	0.7784	0.7566	0.7800	0.6740	0.8022
50	1.0	0.0496	0.0448	0.0526	0.0564	0.0552	0.0402
	1.2	0.1594	0.1664	0.1794	0.1870	0.1410	0.1676
	1.4	0.4078	0.4360	0.4262	0.4438	0.3560	0.4662
	1.6	0.6924	0.7032	0.6942	0.7066	0.5682	0.7306
	1.8	0.8578	0.8740	0.8616	0.8650	0.7796	0.8830
	2.0	0.9494	0.9542	0.9468	0.9512	0.8862	0.9682
100	1.0	0.0470	0.0554	0.0480	0.0536	0.0582	0.0406
	1.2	0.2566	0.2772	0.2398	0.2600	0.2082	0.2844
	1.4	0.6632	0.6812	0.6562	0.6754	0.5416	0.7114
	1.6	0.9036	0.9240	0.9032	0.9040	0.8202	0.9342
	1.8	0.9852	0.9868	0.9866	0.9854	0.9470	0.9922
	2.0	0.9978	0.9990	0.9976	0.9982	0.9856	0.9996

**Table 4:** Power comparisons of the tests in significance level *q* = 0.05.

Weibull							
n=m	β	$t_{N}$	${\hat{\Delta }}_{\mathrm{dil}\;}^{0.5}(X,Y)$	$\hat{\Delta }(n,m)$	$T_{nm}^{2}$	${\hat{D}}_{1}(n,m)$	${\hat{D}}_{2}(n,m)$
25	1.0	0.8770	0.8648	0.8696	0.8784	0.9084	0.7926
	1.2	0.6886	0.6800	0.6756	0.6982	0.7376	0.5948
	1.4	0.4412	0.4370	0.4288	0.4454	0.4792	0.3718
	1.6	0.2182	0.2220	0.2228	0.2404	0.2320	0.1854
	1.8	0.0978	0.0930	0.0848	0.1024	0.0938	0.0842
	2.0	0.2160	0.2024	0.2042	0.1942	0.1582	0.2040
50	1.0	0.9802	0.9766	0.9798	0.9826	0.9926	0.9468
	1.2	0.8888	0.8744	0.8898	0.9000	0.9296	0.7982
	1.4	0.6442	0.6152	0.6434	0.6492	0.7050	0.5556
	1.6	0.3378	0.2986	0.3270	0.3386	0.3682	0.2752
	1.8	0.1198	0.0970	0.1096	0.1144	0.1274	0.0986
	2.0	0.0476	0.0390	0.0442	0.0476	0.0456	0.0454
100	1.0	0.9998	0.9998	0.9998	0.9998	1.0000	0.9988
	1.2	0.9912	0.9888	0.9920	0.9918	0.9940	0.9670
	1.4	0.8946	0.8646	0.8788	0.8774	0.9204	0.7836
	1.6	0.5530	0.5186	0.5184	0.5254	0.5932	0.4172
	1.8	0.1908	0.1686	0.1672	0.1718	0.1968	0.1330
	2.0	0.0568	0.0486	0.0510	0.0482	0.0524	0.0414

**Table 5:** Power comparisons of the tests in significance level *q* = 0.05.

Gamma							
n=m	β	$t_{N}$	${\hat{\Delta }}_{\mathrm{dil}\;}^{0.5}(X,Y)$	$\hat{\Delta }(n,m)$	$T_{nm}^{2}$	${\hat{D}}_{1}(n,m)$	${\hat{D}}_{2}(n,m)$
25	2.0	0.0526	0.0560	0.0574	0.0510	0.0492	0.0494
	2.2	0.0588	0.0568	0.0648	0.0504	0.0544	0.0634
	2.4	0.0816	0.0798	0.0790	0.0714	0.0646	0.0934
	2.6	0.1096	0.0988	0.1044	0.0898	0.0748	0.1302
	2.8	0.1364	0.1322	0.1334	0.1182	0.0846	0.1838
	3.0	0.1696	0.1836	0.1660	0.1520	0.1042	0.2600
50	2.0	0.0484	0.0486	0.0518	0.0488	0.0478	0.0488
	2.2	0.0586	0.0652	0.0684	0.0612	0.0606	0.0696
	2.4	0.0900	0.0998	0.0952	0.0832	0.0706	0.1158
	2.6	0.1340	0.1410	0.1382	0.1314	0.0988	0.2012
	2.8	0.1908	0.2136	0.2096	0.1996	0.1194	0.2976
	3.0	0.2570	0.2756	0.2820	0.2600	0.1482	0.4118
100	2.0	0.0456	0.0452	0.0562	0.0420	0.0502	0.0480
	2.2	0.0676	0.0682	0.0838	0.0626	0.0602	0.0906
	2.4	0.1332	0.1322	0.1360	0.1142	0.0902	0.1840
	2.6	0.2174	0.2144	0.2214	0.2182	0.1334	0.3486
	2.8	0.3252	0.3400	0.3478	0.3120	0.1876	0.5146
	3.0	0.4606	0.4512	0.4658	0.4372	0.2458	0.6570

**Table 6:** Power comparisons of the tests in significance level *q* = 0.05.

Mixture Weibull							
n=m	β	$t_{N}$	${\hat{\Delta }}_{\mathrm{dil}\;}^{0.5}(X,Y)$	$\hat{\Delta }(n,m)$	$T_{nm}^{2}$	${\hat{D}}_{1}(n,m)$	${\hat{D}}_{2}(n,m)$
25	1.0	0.5352	0.5066	0.5380	0.5536	0.5974	0.4788
	1.2	0.3298	0.3260	0.3410	0.3702	0.3906	0.2962
	1.4	0.1832	0.1784	0.1878	0.2032	0.2128	0.1670
	1.6	0.0980	0.1056	0.0988	0.1036	0.1108	0.0996
	1.8	0.0578	0.0646	0.0568	0.0628	0.0556	0.0684
	2.0	0.0480	0.0474	0.0496	0.0432	0.0534	0.0518
50	1.0	0.7666	0.7382	0.7720	0.7598	0.8314	0.6822
	1.2	0.5140	0.4882	0.5352	0.5198	0.6074	0.4442
	1.4	0.2700	0.2568	0.3042	0.2822	0.3602	0.2358
	1.6	0.1266	0.1262	0.1396	0.1316	0.1764	0.1118
	1.8	0.0624	0.0624	0.0748	0.0676	0.0770	0.0594
	2.0	0.0426	0.0508	0.0538	0.0396	0.0552	0.0454
100	1.0	0.9514	0.9402	0.9462	0.9410	0.9702	0.9016
	1.2	0.7814	0.7366	0.7738	0.7564	0.8432	0.6814
	1.4	0.4678	0.4494	0.4810	0.4564	0.5424	0.3798
	1.6	0.2196	0.2162	0.2222	0.2046	0.2442	0.1754
	1.8	0.0950	0.0876	0.0890	0.0878	0.0884	0.0680
	2.0	0.0524	0.0480	0.0504	0.0470	0.0498	0.0526

**Table 7:** Statistical test results for real dataset.

**Test**	$t_{N}$	${\hat{\Delta }}_{dil}^{0.5}(X,Y)$	$\hat{\Delta }(n,m)$	$T_{nm}^{2}$	${\hat{D}}_{1}(n,m)$	${\hat{D}}_{2}(n,m)$
Statistic	32.63350	5.07340	66.54914	32.01251	41.10225	47.00085
*p*-value	0.001112	0.000321	0.000401	0.000600	0.007825	0.000077

## Data Availability

The data generated or analyzed during this study are included in the article.
